# User interfaces for actuated scope maneuvering in surgical systems: a scoping review

**DOI:** 10.1007/s00464-023-09981-0

**Published:** 2023-03-27

**Authors:** Hawa Hamza, Victor M. Baez, Abdulla Al-Ansari, Aaron T. Becker, Nikhil V. Navkar

**Affiliations:** 1 Department of Surgery, Hamad Medical Corporation, Doha, Qatar; 2 Department of Electrical and Computer Engineering, University of Houston, Houston, USA

**Keywords:** Robotic scope control, User interface, Surgical systems, Minimally invasive surgery

## Abstract

**Background:**

A variety of human computer interfaces are used by robotic surgical systems to control and actuate camera scopes during minimally invasive surgery. The purpose of this review is to examine the different user interfaces used in both commercial systems and research prototypes.

**Methods:**

A comprehensive scoping review of scientific literature was conducted using PubMed and IEEE Xplore databases to identify user interfaces used in commercial products and research prototypes of robotic surgical systems and robotic scope holders. Papers related to actuated scopes with human–computer interfaces were included. Several aspects of user interfaces for scope manipulation in commercial and research systems were reviewed.

**Results:**

Scope assistance was classified into robotic surgical systems (for multiple port, single port, and natural orifice) and robotic scope holders (for rigid, articulated, and flexible endoscopes). Benefits and drawbacks of control by different user interfaces such as foot, hand, voice, head, eye, and tool tracking were outlined. In the review, it was observed that hand control, with its familiarity and intuitiveness, is the most used interface in commercially available systems. Control by foot, head tracking, and tool tracking are increasingly used to address limitations, such as interruptions to surgical workflow, caused by using a hand interface.

**Conclusion:**

Integrating a combination of different user interfaces for scope manipulation may provide maximum benefit for the surgeons. However, smooth transition between interfaces might pose a challenge while combining controls.

**Supplementary Information:**

The online version contains supplementary material available at 10.1007/s00464-023-09981-0.

Camera scopes provide surgeons with extensive visualization of internal organs during minimally invasive surgeries. Traditionally, the operating surgeon relies on human assistance to move the camera for optimal views. The human assistant is required to hold the scope in a stable manner so there are no shaky views of the operating field. Long operating times lead to interrupted visualization due to fatigue, tremors, miscommunication, and increased need for cleaning when the lens accidentally touches nearby organs. Poor maneuvering of camera scopes by human assistance can complicate procedures [1].

Camera assistant roles are often assigned to junior surgical residents. Handling the scope requires complex psychomotor skills such as visual-spatial processing, hand–eye coordination, and knowledge of the surgical procedure. Camera navigation skills, such as target centering and smooth movements, are assessed using structured tools or simulators that are designed to differentiate between experienced and inexperienced assistants [2]. The type of skills required vary with the procedure. For example, assistants require more advanced navigation skills for colorectal resections, than for cholecystectomies. As surgeons are fully dependent on camera views during laparoscopic surgeries, any unstable views, smudges on the lens, or collisions with instruments caused by the human assistant can prolong operating time. This may compromise patient safety [3]. Inexperienced assistants may unintentionally rotate the camera scope, thereby affecting the surgeon’s visual perception. This can cause misidentification of anatomic structures and lead to intraoperative injuries [4].

Issues with human camera assistance can be resolved by using scope holders. Camera scope holders that replace human assistance can provide images without the effect of hand tremors. Passive scope holders are maneuvered manually between fixed camera positions. Although clear views without hand tremors are provided, smooth movement of the scope can be challenging [5, 6]. To overcome this, robotic scope holders that allow visual stability and full control by the operating surgeon have become commonplace. Compared to a human camera assistant, an active robotic scope holder provides the operating surgeon with a flexible and steady view, in addition to reducing operating time and cost [5]. Optimal views in human-assisted laparoscopy depend on the training and experience of the assistant, while there is less dependency on these factors in a robot-assisted procedure [7]. Using robotic scope holders offers improved ergonomics for surgeons [8]. While musculoskeletal disorders are prevalent among laparoscopic surgeons due to posture and repetitive movements, reports of physical discomfort, such as wrist, shoulder, back and neck pain, are much lower in robotic surgeries [9].

In robot-assisted surgical procedures, the surgeon controls the slave robot using a master interface. Robotic systems utilize a variety of user interfaces, which include control by foot, hand, voice, head, eyes, and image-based tracking of surgical tools. (Detailed descriptions of each user interface type are presented in the first part of the Results section.) To reduce cognitive load on the surgeon, natural and direct mapping of interface movement with the robotic actuator is required. An ideal interface is intuitive, ergonomic, and user-friendly [10, 11]. Intuitive interfaces help decrease the time required for endoscope tip positioning, which is imperative while performing advanced surgical interventions [12].

Surgical robotic systems (and hence the user interfaces to control them) vary as per the intervention site. Surgical sites close to an entry port may only require rigid or semi-rigid scopes for visualization. However, complex procedures in the gastrointestinal tract, such as endoscopic submucosal dissection (ESD), require robotically actuated flexible scopes for manipulation and optimal positioning [13]. Biopsies of peripheral pulmonary lesions benefit from robotic bronchoscopy, which allows scope navigation for direct visualization through bronchi that branch at different angles, and become progressively smaller deeper in the lungs [14]. Improved surgical precision that allows fine dissection makes robot assistance favorable for urological and colorectal surgeries.

To our knowledge, current literature does not provide a detailed review of the different scope user interfaces in robotic surgery. This review aims to provide an overview of user interfaces for robotically actuated camera scopes. The Results section describes the common user interfaces used by robotic systems for visualization during surgery. It also covers the different robotic surgical systems that actuates scope. It further provides mapping of user interfaces with the robotic systems as well as the surgeries performed under different specialties. The Discussion section describes the evolution of user interfaces over time. A comparison of key features of different user interfaces are also presented.

## Methods

The review follows the Preferred Reporting Items for Systematic Reviews and Meta-Analysis extension for Scoping Reviews (PRISMA-ScR) guidelines [15]. An extensive search of scientific literature was conducted using PubMed and IEEE Xplore databases to identify articles describing user interfaces for robotic scope control in surgery. The search strategy for PubMed is given in Supplementary Content 1. Additional records were identified through thorough citation searches, websites, and patents. A total of 720 records were screened. Articles related to surgical systems using actuated scopes with user interfaces published between 1995 and 2022 were included. The records were screened using Rayyan app (https://www.rayyan.ai/). Duplicate reports, non-robotic passive systems, soft robots, systems not related to endoscopic or laparoscopic visualization, and papers not in English were excluded. Data extracted from the records were categorized into user interfaces and types of robotic systems. Additional citations were also used (such as company websites) to provide references for the technical specifications of the robotic systems. In addition, papers comparing different user interfaces were also identified.

## Results

**Fig. 1:**
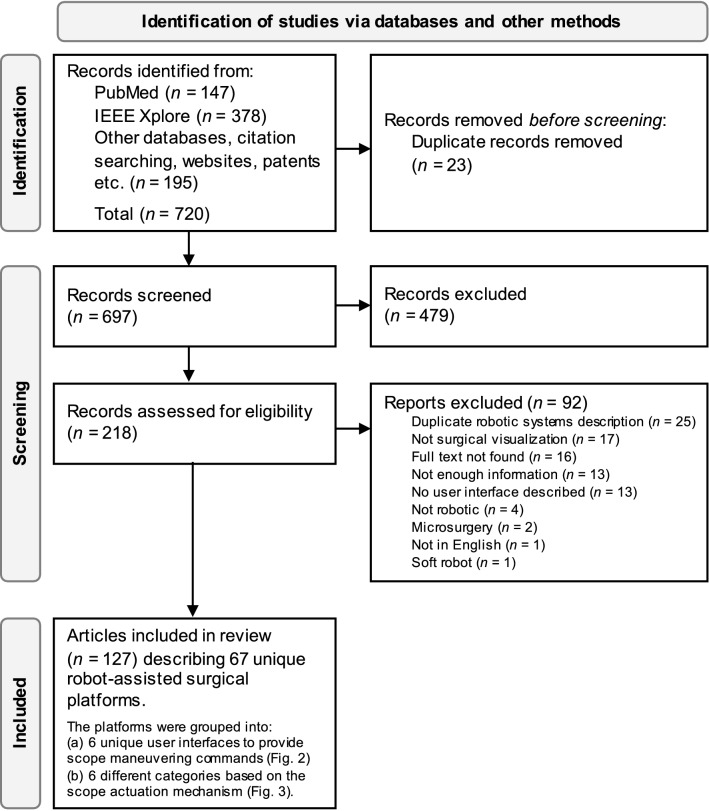
Record identification and screening flowchart

**Fig. 2:**
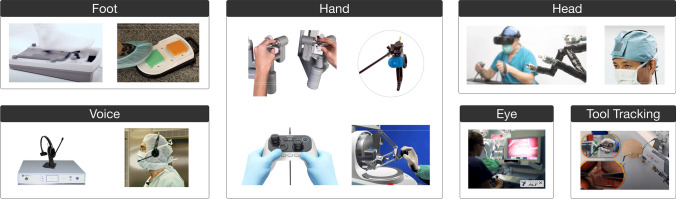
Examples of interfaces to control scopes used in robot-assisted surgeries

**Fig. 3:**
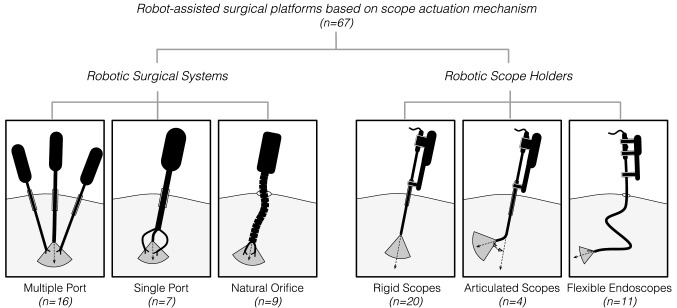
Categories of robotic systems for visualization during surgery

A total of 127 articles describing 67 different robot-assisted surgical platforms were included in the review after identifying and screening (Fig. 1). The platforms were grouped into: (a) 6 unique user interfaces to provide scope maneuvering commands (Fig. 2) and (b) 6 different categories based on the scope actuation mechanism (Fig. 3). Various characteristics of each robotic system, including (a) visualization type (stereo vision, high-definition, camera size, resolution), (b) degree(s) of freedom (DOF), (c) manipulation type (insertion, retraction, pan, tilt, rotate), (d) actuation method (motor, pneumatically driven), (e) control type (teleoperated, cooperative), (f) control interface, (g) development stage (commercial, research), (h) year, and (i) clinical application were also extracted.

Primary findings of the searches conducted are presented in the three subsequent sections. The first section describes the user interfaces for actuated scope control. The second section presents robot-assisted surgical platforms based on scope manipulation. A more detailed account of user interfaces used with different robot-assisted surgical platforms and in different surgeries is presented in the third section.

### User interfaces to provide scope maneuvering commands

Robotic systems increase the performance of camera scopes by filtering tremors and translating precise movements. Intuitive user interfaces have been developed for control of robotic systems. These can be categorized by mode of input, which includes control by foot, hand, voice, head, eyes, and image-based tracking of surgical tools, as illustrated in Fig. 2.

#### Foot control

Foot pedals are often used as a clutch to activate scope control using handles such as finger loops or joystick [16]. The camera position is fixed unless the clutch is engaged. Foot pedals may also act as an independent control, such as the consoles developed by Yang et al. [17] and Huang et al. [16], where the novel foot interface controls the scope in four degrees of freedom (DOF). Foot control frees the hands for controlling surgical instruments. However, the buttons pressed by the foot may distract the surgeon’s attention, as they look down to differentiate the correct pedal from the ones used for operating an electric knife or other instruments [18].

#### Hand control

The types of hand control devices that have been adopted by commercially available systems include joysticks, buttons, finger loops, touch pads, and trackballs. These allow operating surgeons to have independent control on the visualization without relying on human assistance. The application of this type of interfacing is limited because surgeons cannot simultaneously operate the scope and their instruments [16]. Surgical flow is interrupted as the operating surgeon switches between control of surgical instrument and camera scope. Additionally, pain in the fingers and thumb is commonly reported for robotic surgeries during prolonged use [9].

#### Voice control

In systems controlled by voice, the surgeon speaks out commands such as “up”, “down”, “in”, “out” etc., to move camera scopes. Manipulating camera scopes using voice control mimics the default communication method used between operating surgeon and assistant, and there is no physical fatigue [19]. Noise in the background, however, can potentially affect voice recognition accuracy. Repetition of voice commands causing considerable delay in scope movement make it unfavorable for surgeons [20]. The typical task time for voice control is 2 s [21].

#### Head control

Head motion tracking provides a non-verbal intuitive control method using the surgeon’s head position as input data. Recognition of facial gestures [22] and use of head mounted displays [23] allows smooth scope control without discontinuing surgical tasks. However, it can be challenging to intuitively control the depth of the endoscope using head movements [24].

#### Eye tracking

Eye tracking involves navigating the scope using eye gaze control by measuring reflections in the cornea. Although eye tracking methods free up hands for surgical instruments, they can be considered distracting. In a study [25] reporting surgeon’s opinion on interfaces, 3 out of 5 surgeons rated eye tracking unfavorably.

#### Tool tracking

Tool tracking uses image analysis that continuously detects the surgical instruments when activated and controls the scope position accordingly. Automatic view centering and zoom adaption is possible with the computer-based instrument tip tracking system. However, surgeons might have different priorities in terms of what they want to see while using instrument tracking [26]. This control can be challenging for tasks without surgical tools.

### Robot-assisted surgical platforms based on scope manipulation

This section presents the robot-assisted surgical platforms that utilize aforementioned user interfaces to visualize the operative field during surgery. As depicted in Fig. 3, two main categories were used: (i) robotic surgical systems (grouped based on access to surgical site: multiple port, single port, and natural orifice), and (ii) robotic scope holders (grouped based on flexibility of scope used: rigid, articulated, and flexible endoscopes).

#### Robotic surgical systems for multiple-port surgeries

As opposed to conventional laparoscopic surgery, robotic surgery provides enhanced visualization, dexterity, and ergonomics. Systems made for multiple-port surgeries utilize several incisions to gain access to the target area [27]. A surgeon console, either closed or open, with controllers is employed to teleoperate the robotic arm holding the camera scope. The surgeon may also switch ports over the course of the procedure. Robotic systems for multiple-port surgeries (Table 1), such as the da Vinci Xi (Intuitive Surgical Inc., USA) and Senhance (Asensus Surgical, USA), are utilized for a wide variety of clinical applications such as colorectal, general, gynecological, thoracic, and urological surgeries [28–30].

**Table 1:** Robotic surgical systems for visualization in multiple-port surgeries, by year

Name	Visualization type	DOF^a^		Manipulation type	Actuation method	Control type^b^ & level of automation^c^	Control interface	Development stage	Year	Clinical application
		Camera	Total							
ARTEMIS (Karlsruhe Research Center, Germany)^d^ [31]	Three-dimensional (3D) endoscopic vision system	4	6	Steerable & rotation of instruments 90° bending angle	Electromotors	Teleoperated Master–slave	Open surgeon workstation—joystick control for endoscope, two master arms	Research prototype (animal studies) (defunct)	1999	*Surgery—minimally invasive* Cardiac surgery
ZEUS Robotic Surgical System (Computer Motion Inc., USA)^e^ [32]	10 mm 3D laparoscope	4	6	One-way articulating tips	Motor	Teleoperated Master–slave	Open surgeon console Voice-activated camera system	Commercial (defunct)	2001 (FDA)	*Surgery—minimally invasive* General surgery (gastrectomy, cholecystectomy)
da Vinci Xi (Intuitive Surgical Inc., USA) [29, 33]	3D high definition (HD) 8 mm 30° endoscope Fluorescence imaging	–	7	Insertion, retraction	Cable-driven	Teleoperated Master–slave	Closed surgeon console—hand controllers (finger loops), foot pedal (clutch)	Commercial	2014 (FDA)	*Surgery—minimally invasive* Cholecystectomy, prostatectomy, hysterectomy, colorectal cancer surgery, cardiothoracic surgery, head & neck surgery
Micro Hand S (Tianjin University, China) [34]	3D camera	–	7	360° rotation	Cable-driven	Teleoperated Master–slave	Open surgeon console—hand control	Commercial	2014 (China)	*Surgery—minimally invasive* General & colorectal surgery (total mesorectal excision, sigmoidectomy)
Senhance Surgical System (Asensus Surgical USA, Inc.)^f^ [35–37]	3D HD vision, fluorescence visualization	–	7	Insertion, retraction, pan, zoom	Electrical motor	Teleoperated Master–slave	Open surgeon console—track pad & handles 3D glasses, eye-tracking	Commercial	2017 (FDA)	*Surgery–minimally invasive* Colorectal, gynecological, general, urological, thoracic
Revo-i (meerecompany, South Korea) [37–39]	3D HD	–	7	Zoom, rotate	Electrical motor	Teleoperated Master–slave	Closed surgeon console–precision grip finger controls & foot pedal (clutch)	Commercial	2017 (Korea)	*Surgery—minimally invasive* Urology, general, obstetrics & gynecology
Bitrack (Rob Surgical, Spain) [29, 40]	3D HD	–	7	–	–	Teleoperated Master–slave	Open surgeon console with hand controls 3D glasses Haptic feedback	Research prototype (animal studies)	2018	*Surgery—minimally invasive* General, urology, colon & rectal, gynecology, thoracic, renal & hepatic
avatera (avateramedical, Germany) [41, 42]	3D HD vision	–	7	–	–	Teleoperated Master–slave	Closed control unit with slender eyepiece, handle, footswitch	Commercial	2019 (CE)	*Surgery—minimally invasive* Urology (removal of prostate & kidney tumors), gynecology
Versius (CMR Surgical, UK) [43]	3D HD camera system	–	7	–	Electrical motor	Teleoperated Master–slave	Open operator console with joystick controllers 3D glasses	Commercial	2019 (CE)	*Surgery—minimally invasive* Gynecologic, colorectal, renal, head & neck, upper gastrointestinal
hinotori™ (Medicaroid Corporation, Japan) [44, 45]	3D vision	4	8	–	–	Teleoperated Master–slave	Semi-open surgeon cockpit—3D viewer, hand control, foot pedal (clutch)	Commercial	2020 (Japan)	*Surgery—minimally invasive* Prostatectomy
Dexter (Distalmotion, Switzerland) [46, 47]	–	–	7	In/outward, up/downward, left/right, rotational, pitch, yaw, open/close	Cable-driven	Teleoperated Master–slave	Open surgeon console with handle grip	Commercial	2020 (CE)	*Surgery—minimally invasive* Gynecology surgery (hysterectomy)
Jo, Kim [48] (Seoul National University, South Korea) [48]	3D endoscope	4	–	Up/down, right/left, roll	Cable-driven	Teleoperated Master–slave	VR headset Head tracking	Research	2020	*Surgery—minimally invasive* Laparoscopic surgery
Toumai Endoscopic Surgical System (MicroPort MedBot, China) [49]	3D view	–	7	–	–	Teleoperated Master–slave	Closed surgeon console with hand controls, foot pedal (clutch)	Commercial	2021 (China)	*Surgery—minimally invasive* Urology (prostatectomy, nephrectomy)
SHURUI (Beijing Surgerii Technology Co. Ltd., China) [27, 50, 51]	3D stereo vision 10 mm diameter 60 fps 1280 × 720	6	–	Tip deflection	Cable-driven	Teleoperated Master–slave	Open surgeon console—hand controllers (customized Geomagic TouchX devices)	Research prototype (human clinical trials)	2021	*Surgery—minimally invasive* Radical resection of sigmoid colon cancer, gynecologic surgeries (radical nephrectomy, partial bladder resection, thoracoscopic mediastinal lymph node dissection in porcine models)
Hugo RAS system (Medtronic, USA)^g^ [52–54]	3D visualization	–	7	–	Cable-driven	Teleoperated Master–slave	Open surgeon console 3D HD vision Hand grip controllers Foot pedal (clutch)	Commercial	2021 (CE)	*Surgery—minimally invasive* Urologic (prostatectomy) and gynecologic procedures
SSI Mantra (SS innovations, India) [55]	3D HD chip-on-tip articulating scope	4	–	Four-way articulation	–	Teleoperated Master–slave	Open surgeon console with hand control (mini joystick), foot pedal (clutch)	Commercial	2022 (India)	*Surgery—minimally invasive* Urology, general surgery, gynecology, thoracic, cardiac, head & neck

^a^DOF refers to degree(s) of freedom

^b^Control type: Teleoperated, cooperative, autonomous

^c^Level of automation: Master-slave, semi-autonomous, autonomous

^d^ARTEMIS used FIPS robotic scope holder. It was not developed further

^e^ZEUS used AESOP robotic scope holder. Computer Motion was acquired by Intuitive Surgical

^f^Senhance was formerly known as Telelap Alf-X. Asensus Surgical US, Inc. was previously known as TransEnterix, Inc.

^g^Hugo RAS incorporates MiroSurge (German Aerospace Center DLR, Germany)

#### Robotic surgical systems for single-port surgeries

Compared to multiple-port procedures, single-port surgeries reduce invasiveness and significantly benefit patients with less scarring, low recovery time and reduced postoperative pain [56]. Robotic systems developed for single-incision laparoscopic surgeries, as detailed in Table 2, usually have a single arm with multiple instruments and a scope for visualization that extends outwards. The incision may be of different sizes depending on the system used and the procedure. Single-port surgery may prove challenging for the surgeon due to poor ergonomics. To avoid collision, distally actuated arms that achieve triangulation of the instruments around the target organ are often required [57]. Much like the ones for multiple-port surgeries, these systems utilize either closed or open surgeon console with controllers to manipulate the robotic arm. The da Vinci SP (Intuitive Surgical Inc., USA) has US Food and Drug Administration (FDA) approval for urologic and transoral otolaryngology procedures. Other platforms under development target gynecological and general surgery applications.

**Table 2:** Robotic surgical systems for visualization in single-port surgeries, by year

Name	Visualization type	DOF		Manipulation type	Actuation method	Control type & level of automation	Control interface	Development stage	Year	Clinical application
		Camera	Total							
da Vinci SP Surgical System (Intuitive Surgical Inc., USA) [36, 58]	12 × 10 mm articulating camera	–	7	Double articulating (wrist & elbow) endoscope 360° rotation	Cable-driven	Teleoperated Master–slave	Closed surgeon console—hand controllers (finger loops), foot pedal	Commercial	2014 (FDA)	*Surgery—minimally invasive* Urologic (prostatectomy, cystectomy, nephrectomy, pyeloplasty), transoral otolaryngology surgeries, transanal total mesorectal excision in human cadaveric model
SurgiBot, (TransEnterix, Inc., USA)^a^ [30]	3D HD visualization	–	6	Retraction	–	Teleoperated Master–slave	Patient-side hand controller with knobs	Research prototype (towards commercialization)	2015	*Surgery—minimally invasive* Abdominal surgery General and urology procedures
SJTU unfoldable robotic System (SURS) (Shanghai Jiao Tong University, China) [59]	3D vision unit 640 × 480	3	6	Bending & translation	Motor-driven actuation rods	Teleoperated Master–slave	Hand control (Phantom Omni devices)	Research prototype (lab studies)	2015	*Surgery—minimally invasive* Single-port laparoscopic procedures
Vicarious surgical system (USA) [60, 61]	Two cameras 3D HD 360° visibility, panoramic view	2	9	Pan, tilt 180° swivel	Cable-driven	Teleoperated Master–slave	Open surgeon console with head mounted display	Research prototype (under development)	2017	*Surgery—minimally invasive* Ventral hernia repair
SPAS robotic system (National University of Singapore, Singapore) [62, 63]	5.5 mm diameter 1280 × 720 resolution	2	5	–	Tendon-sheath mechanism	Teleoperated Master–slave	Hand control (two geomagic touch haptic devices)	Research prototype (design concept)	2019	*Surgery—minimally invasive* Appendectomy, nephrectomy Oncology—treatment of giant cell tumor
Enos Surgical System (Titan Medical Inc., Canada)^b^ [28, 37, 64, 65]	2D & 3D HD	–	6	Elevate, tilt, pan	Electrical motor	Teleoperated Master–slave	Open surgeon console—hand controllers & foot pedal (clutch)	Research prototype (animal & human cadaver studies)	2020	*Surgery—minimally invasive* Cholecystectomy, fundoplication, future gynecologic application
MIRA (Virtual Incision, USA) [66]	Full HD (1080p /60 Hz)	–	7	Articulating flex tip	–	Teleoperated Master–slave	Open surgeon console—hand controllers, foot pedals, touchscreen Haptic feedback	Research prototype (FDA clinical trials)	2022 (FDA IDE)	*Surgery—minimally invasive* Bowel resection procedures

^a^SurgiBot was built on Single Port Instrument Delivery Extended Research (SPIDER). SurgiBot assets were later sold to Great Belief International Limited (GBIL), China for commercialization. TransEnterix, Inc. is currently known as Asensus Surgical US, Inc.

^b^Enos was previously known as Single Port Orifice Robotic Technology (SPORT)

#### Robotic surgical systems for natural orifice procedures

Further minimizing surgical aggressiveness, robotic systems for natural orifice procedures approach the site of interest through the natural openings in the body such as the mouth or anus [67]. This is especially beneficial when the patient has a compromised immune system. The robot consists of a highly flexible and dextrous arm that can be steered towards intricate structures. An open surgeon console or a bed-side controller is used to manipulate the arm, and correspondingly the camera. Table 3 describes robotic systems used for transoral applications such as vocal cord lesion resection and bronchoscopy, as well as colorectal surgeries. Systems aimed for endoscopic submucosal dissection (ESD) in the gastrointestinal tract and ear, nose, throat (ENT) surgeries are under development.

**Table 3:** Robotic surgical systems for visualization in natural orifice procedures, by year

Name	Visualization type	DOF		Manipulation type	Actuation method	Control type & level of automation	Control interface	Development stage	Year	Clinical application
		Camera	Total							
Flex system (Medrobotics Corp., USA) [29, 68, 69]	3D HD Dual 1920 × 1080 pixel 80° field of view	–	–	180° articulation, horizontal, vertical, rotation, zoom	Cable-driven	Teleoperated Master–slave	Open console Single-port control joystick	Commercial	2015 (FDA)	*Surgery—minimally invasive* Transoral surgery (oropharyngeal, hypopharyngeal, laryngeal procedures) Obstetric/gynecologic applications
MONARCH platform (Auris Health, Inc., USA)^a^ [14, 70–73]	660p x central airways & periphery vision	10	–	Insertion, retraction, articulation 180° in all direction	Cable-driven	Teleoperated Master–slave	Hand-held controller (joysticks & buttons)	Commercial	2018 (FDA)	*Investigational procedure* Robotic bronchoscopy for peripheral pulmonary lesion biopsy Surgery—minimally invasive Urology—percutaneous nephrolithotomy
STRAS (ICube^b^) [13, 16]	–	2	10	Rotation, deflection, translation	Motor (tendon-driven)	Teleoperated Master–slave	Handle shaft on L-shaped bracket, two small four-way finger joysticks to operate endoscope	Research prototype (animal studies)	2018	*Surgery—minimally invasive* Treatment of tumor in rectum and sigmoid colon Gastrointestinal tract surgery Endoscopic submucosal dissection (ESD) in animal model
*i*^*2*^*Snake* (Hamlyn Centre, UK) [74]	3 mm 640 × 480 pixels	–	7	–	Tendon driven actuated by EC motors	Teleoperated Master–slave	Hand-held gripper Foot pedal for switching modes	Research prototype (lab studies)	2018	*Surgery—minimally invasive* Transoral surgery Tumor resection, sleep-apnea surgery
Ion endoluminal system (Intuitive Surgical Inc., USA) [75–77]	Removable vision probe 90° field of view 0° direction of view	–	–	180° in all direction (pitch & yaw)	Electromechanically (servo/stepper motors & software)	Teleoperated Master–slave	Hand control (trackball & scroll wheel)	Commercial	2019 (FDA)	*Investigational procedure* Minimally invasive peripheral lung biopsy (bronchoscopy)
Endoscopic Therapeutic Robot System (ETRS) (Kyushu Institute of Technology, Japan)^c^ [78]	120° field of view	4	–	Up/down & left/right angulation, insertion/retraction, rotation	Motor	Teleoperated Master–slave	Hand controls (Geomagic Touch)	Research prototype (animal studies)	2019	*Surgery—minimally invasive* Endoscopic submucosal dissection (ESD) in porcine model
K-FLEX (EasyEndo Surgical, Korea) [79]	High definition	4	14	Deflection, translation, rotation	Wire cable & motor	Teleoperated Master–slave	Hand interface switched by foot clutch	Research prototype (ex vivo porcine study)	2020	*Surgery—minimally invasive* Possible application for gastrointestinal tract, ENT surgeries
Three-Limb Robotic System (Nanyang Technological University, Singapore)^d^ [16, 80, 81]	120° field of view 0° forward viewing	4	13	Up/down, left/right, in/out, rotation	Tendon-sheath mechanism & motors	Teleoperated Master–slave	Open master console Two hand interfaces One foot interface to control endoscope	Research prototype (ex vivo porcine study)	2021	*Surgery—minimally invasive* Transoral robotic surgery Gastrointestinal tract surgery Endoscopic resection
Endoluminal Surgical System (EndoQuest Robotics, USA)^e^ [82, 83]	3.7 mm HD robotic camera	–	7	Advanced flexibility & dexterity	–	Teleoperated Master–slave	Open surgeon console—hand controllers & foot pedal (clutch)	Research prototype (clinical trial)	2021	*Surgery—minimally invasive* Transanal endoluminal procedures; colorectal endoscopic submucosal dissection (ESD)

^a^Auris Health previously acquired Hansen Medical, manufacturer of Magellan & Sensei robotic systems. Auris Health was later acquired by Johnson & Johnson, which plans to build Ottava.

^b^STRAS is a robotic version of Anubiscope (IRCAD & KARL STORZ Endoskope)

^c^The endoscope is controlled by endoscopic operation robot (EOR)

^d^Nanyang Technological University has also produced the robotic system EndoMaster (EndoMaster Pte Ltd., Singapore). However, it requires manual operation of the endoscope

^e^Endoluminal Surgical System was previously known as ColubrisMX ELS System

#### Robotic scope holders for rigid scopes

Minimally invasive surgeries employ rigid scopes for visualization that is either zero-degree which is forward-viewing or angulated that provides a wide range of view. Robotically actuated scope holders, which are used to hold and maneuver rigid scopes, provide a tremor-free stable view that is directly controlled by the operating surgeon. It eliminates the need to communicate desired scope position changes to an assistant [84]. Several holders have been developed for rigid scopes, with AESOP (Computer Motion, USA) being one of the earliest robotic scope holders using hand, foot, and voice control. As described in Table 4, they are used extensively in general, urology, gynecology, and colorectal surgeries. SOLOASSIST II (AKTORmed, Germany) has applications in transoral thyroid surgeries as well.

**Table 4:** Robotic scope holders for rigid scopes, by year

Name	DOF	Manipulation type	Actuation method	Control type & level of automation	Control interface	Development stage	Year	Clinical application
AESOP (Computer Motion Inc., USA)^a^ [85]	4	Three rotations and insertion depth	Motor	Teleoperated Master–slave	Hand control joystick, voice commands, foot pedal control	Commercial (defunct)	1994 (FDA)	*Surgery—minimally invasive* Thoracic surgery
FIPS (Karlsruhe Research Center, Germany) [86, 87]	4	3 revolute & 1 prismatic joint Up/down, left/right, in/out, rotate	Motor	Teleoperated Master–slave	Finger-ring joystick Voice control	Research prototype (animal studies) (defunct)	1999	*Surgery—minimally invasive* Cholecystectomy
FAce MOUSe (Osaka University, Japan) [22]	3	Up/down, left/right, insertion/retraction	Motor	Teleoperated Master–slave	Facial motion (image-based system), voice commands	Research prototype (ex vivo & in vivo trial)	2003	*Surgery—minimally invasive* Cholecystectomy
LapMan (Medsys, Belgium) [88–90]	3	In/out, right/left, up/down	Motor	Teleoperated Master–slave	Hand control joystick & remote-controlled keypad	Commercial	2003 (FDA)	*Surgery—minimally invasive* Gynecology surgery
Naviot (Hitachi, Japan) [91]	–	Zoom, vertical and horizontal directions	Motor	Teleoperated Master–slave	Hand controller with two buttons	Commercial	2008 (Japan)	*Surgery—minimally invasive* Thoracoscopic surgery (anatomical pulmonary resection) Cholecystectomy
ViKY (EndoControl, France) [92–95]	3	Up-down, left–right, forward–backward	Motor	Teleoperated & cooperative, master–slave & semi-autonomous (detection & tracking of instrument using image analysis)	Voice control, instrument tracking	Commercial	2008 (FDA)	*Surgery—minimally invasive* Radical prostatectomy, gynecology, abdominal, thoracoscopic surgery
FreeHand (FreeHand Surgical, UK)^b^ [86, 96]	3	Pan, tilt, zoom	Motor	Teleoperated Master–slave	Headset with footswitch (to engage movement)	Commercial	2009 (FDA)	*Surgery—minimally invasive* General, gynecology, urology, thoracic surgeries
EVOLAP (Université catholique de Louvain, Belgium) [97, 98]	2	–	Motor	Teleoperated Master–slave	Miniature hand joystick	Research prototype (in vivo trial)	2013	*Surgery—minimally invasive* Gynecology (salpingectomy)
RoboLens (Sina Robotics & Medical Innovators Co., Ltd., Iran) [20, 99–101]	4	Up/down, left/right, in/out, rotation	Motor	Teleoperated & Cooperative Master–slave & Semi-autonomous (Tracking surgical instruments)	Six-button foot pedal Touch screen keypad Voice commands Surgical instruments tracking (image processing)	Commercial	2015 (Iran)	*Surgery—minimally invasive* Cholecystectomy Ovarian cystectomy
AutoLap (MST Medical Surgery Technologies, Israel)^c^ [8, 86, 102, 103]	–	Up/down, left/right, zoom in/out Tracking designated tool	Motor	Teleoperated & Cooperative Master–slave & Semi-autonomous (Automatic view centering, zoom adaption, camera horizon correction)	Joystick (Image analysis and computer-based instrument recognition)	Commercial	2016 (FDA)	*Surgery—minimally invasive* General, gynecology, urology procedures
EMARO (Riverfield Inc., Japan) [86, 104–106]	4	Pan, tilt, zoom, roll	Pneumatically driven	Teleoperated Master–slave	Head sensor, foot pedal (clutch)	Commercial	2015 (Japan)	*Surgery—minimally invasive* Inguinal hernia repair
MTG-H100 (HIWIN Technologies Corp., Taiwan) [23, 107, 108]	3	Zoom in/out, upward/downward, right/left	Motor	Teleoperated Master–slave	Controller with foot pedals Head mounted display & speech controller proposed	Commercial	2017	*Surgery—minimally invasive* General, urology, gynecology, colon & rectal surgeries
Cirq (Medineering, Germany)^d^ [109]	7	Forward/backward, left/right, up/down, pivot point rotation	Motor	Teleoperated Master–slave	Foot pedal controller with joystick	Commercial	2017 (CE)	*Surgery—minimally invasive* Transnasal sinus and skull base surgery
EinsteinVision 3.0 (Aesculap AG, Germany) [110, 111]	–	–	Motor	Teleoperated Master–slave	Remote hand control button interface	Commercial	2017	*Surgery—minimally invasive* Abdominal surgery (upper gastrointestinal procedure) Gynecology surgery
SOLOASSIST II (AKTORmed GmbH, Germany) [112–115]	3	Up/down, left/right Zoom in/out	Electrical motor^e^	Teleoperated Master–slave	Voice control, joystick	Commercial	2018 (FDA)	*Surgery—minimally invasive* General, urology, gynecology, thoracic, cardiac surgeries Transoral endoscopic thyroid surgery
ROSA ONE Brain (Zimmer Biomet, USA) [116]	6	–	–	Cooperative & semi-autonomous (force torque sensor, preoperative or intraoperative planning values)	Touchscreen Foot pedal (for activation) Haptic technology	Commercial	2019 (FDA)	*Investigational procedure* Ventricular endoscopy Transnasal endoscopy *Surgery—minimally invasive* Neurosurgery (brain and spine)
De Pauw, Kalmar [117] (Ghent University, Belgium)	–	Zoom in/out	Electromotor	Master–slave	Single-hand control (thumb lever)	Research prototype (cadaveric trial)	2020	*Surgery—minimally invasive* Colorectal surgery (single-port rectopexy)
Yang, Udatha [17] (Monash University, Australia)	4	Left/right Forward/backward Insertion/withdrawal Rotation	–	Teleoperated Master–slave	Foot interface	Research prototype (lab studies)	2020	*Surgery—minimally invasive* Laparoscopy
FREEDOM (The Chinese University of Hong Kong) [118, 119]	3	Horizontal/vertical, pitch/yaw, translation	Motor	Teleoperated Master–slave	Foot control	Research prototype (clinical trials)	2020	*Surgery—minimally invasive* Endoscopic sinus surgery
Avellino, Bailly [120] (Sorbonne Université, France)	–	Left/right	Cable-driven	Teleoperated & Cooperative Master–slave & Semi-autonomous	Hand manipulation, joystick, tool tracking, posture/head tracking	Research prototype (lab studies)	2020	*Surgery—minimally invasive* Urology, gynecology surgery Bed-side robotic surgery

^a^AESOP is no longer commercialized. Computer Motion was taken over by Intuitive Surgical

^b^FreeHand (previously Prosurgics, UK) replaced EndoAssist / EndoSista (Armstrong Healthcare, UK)

^c^TransEnterix Inc. previously acquired MST Medical Surgery Technologies. AutoLap assets were later sold to Great Belief International Limited (GBIL), China [51]

^d^Medineering was acquired by Brainlab, Germany

^e^Previous generation of the system (SOLOASSIST) was fluid actuated

#### Robotic scope holders for articulated scopes

Articulated scopes have a flexible distal end that improves visualization around complex anatomy. Such scopes reduce the chance of interference with surgical instruments inserted through the same port. Research prototypes of scope holders described by Li et al. [121] and Huang et al. [26] aim towards thoracic surgery applications (Table 5). These research prototypes tend to use a variety of different control interfaces for scope manipulation.

**Table 5:** Robotic scope holders for articulated scopes, by year

Name	DOF	Manipulation type	Actuation method	Control type & level of automation	Control interface	Development stage	Year	Clinical application
Cardioscope (The Chinese University of Hong Kong, China) [121, 122]	–	180° bending with controllable length	Wire-driven flexible mechanism	Cooperative Master–slave	Control body with handle and actuation module	Research prototype (ex vivo & in vivo tests)	2016	*Surgery—minimally invasive* Cardiac surgery (single hole)
Omori, Arai [123] (Chuo University, Japan) [123, 124]	3	Pan-tilt, pitch-yaw, zoom in/out	–	Teleoperated Master–slave	Head-mounted interface detecting jaw movements	Research prototype (lab studies)	2021	*Surgery—minimally invasive* Cholecystectomy
PliENT (Robotics, Automation and Mechatronics Group, Belgium) [125]	6	Distal end steering Bend up to 93°	Pneumatic	Teleoperated Master–slave	Single-handed button interface (Adafruit keypad)	Research prototype (concept design)	2022	*Surgery—minimally invasive* Endoscopic maxillary sinus surgery
Augmented Reality Visualizing Robotic Stereo Flexible Endoscope (ARSFE) (The Chinese University of Hong Kong, China) [26, 126]	6	Rotation, depth, view centering	Cable-driven	Autonomous Fully autonomous (Image moment-based visual servoing method)	Tracking surgical instrument or surgeon’s head (Foot pedal to activate different modes)	Research prototype (lab & animal studies)	2022	*Surgery—minimally invasive* Thoracic surgery

#### Robotic scope holders for flexible endoscopes

Flexible endoscopes are highly dexterous and heavily used in gastroscopy and colonoscopy procedures. Complex movements are required when compared to rigid scopes [127]. Few robotic scope holders have been developed for forward-viewing flexible endoscopes (Table 6). Certain motions, such as rotation, are still controlled manually in some of these systems. Majority of the scope holders are exclusively used for colonoscopy and gastroscopy. The Avicenna Roboflex (ELMED Medical Systems, Türkiye) has applications in urology as well.

**Table 6:** Robotic scope holders used for flexible endoscopes, by year

Name	DOF	Manipulation type	Actuation method	Control type & level of automation	Control interface	Development stage	Year	Clinical application
NeoGuide endoscopy system (NeoGuide Systems Inc., USA)^a^ [128–131]	–	Steering with natural loop maintained	Electromechanical motor	Teleoperated Semi-autonomous	Open console system with joystick (Computer console shapes according to natural loops of colon)	Commercial (defunct)	2007 (FDA)	*Investigational procedure* Colonoscopy
Endotics endoscopy System (Era Endoscopy SRL, Italy) [130, 132, 133]	–	Steering	Pneumatic	Teleoperated Semi-autonomous (Self-propelling)	Workstation with hand-held console	Commercial	2009 (CE)	*Investigational procedure* Colonoscopy
Endodrive (ECE Medical systems, Germany) [84, 134, 135]	–	Shaft insertion, retraction	Electro-mechanical	Teleoperated Master–slave	Foot pedal	Commercial	2010	*Investigational procedure* Colonoscopy, biopsy *Surgery—minimally invasive* Polypectomy
Avicenna Roboflex (ELMED Medical Systems, Türkiye) [136, 137]	–	Forward/backward, insertion/retraction, rotation, deflection	Motor	Teleoperated Master–slave	Console with touchscreen and hand manipulator controls (wheel & joystick)	Commercial	2013 (CE)	*Investigational procedure* Flexible ureterorenoscopy *Surgery—minimally invasive* Urology (retrograde intrarenal surgery)
Teleflex (University of Twente, Netherlands) [10, 138]	4	Distal tip actuation (up/down, left/right) Shaft translation, rotation	Motor	Teleoperated Master–slave	Hand control Head movements	Research prototype (lab studies)	2013	*Surgery—minimally invasive* Transoral gastrointestinal procedures
Aer-O-Scope (GI View, Israel) [139–141]	–	Steering	Pneumatic	Teleoperated Semi-autonomous (Self-navigation)	Open workstation with full joystick control (Computer algorithm adjusts pressure)	Commercial	2016 (FDA)	*Investigational procedure* Colonoscopy
invendoscopy E200 System (invendo medical, Germany)^b^ [29, 142, 143]	–	180° tip deflection Tip steering, shaft translation	Electromechanical motor	Teleoperated Master–slave	Open invendo ScopeController with hand-held joystick	Commercial	2016 (FDA)	*Surgery—minimally invasive* Colonoscopy Polypectomies
Gastroscope intervention mechanism (GIM) (Chinese Academy of Sciences, China) [144]	2	Push-pulling, rotating	Pneumatic pressure	Teleoperated Master–slave	Hand control joystick	Research prototype (in vivo live animal studies)	2017	*Investigational procedure* Gastroscopy
Endoscopic operation robot (EOR) (Kyushu Institute of Technology, Japan) [145, 146]	4	Up/down & left/right angulation, insertion/retraction, rotation	Motor	Teleoperated Master–slave	Hand control mini-joystick & knobs	Research prototype (lab studies)	2018	*Investigational procedure* Colonoscopy *Surgery—minimally invasive* Endoscopic submucosal dissection (ESD) in porcine model
Robotic-assisted flexible endoscope (RAFE) (Kyushu University, Japan) [127]	4	Up-down, right-left, back–forth, twisting	Motor	Teleoperated Master–slave	One-handle master controller	Research prototype (porcine model)	2018	*Surgery—minimally invasive* Endoscopic submucosal dissection (ESD)
Sivananthan, Kogkas [147] (NHS & Imperial College London, UK)	–	Steering, advancement, withdrawal, retroflexion	Motor	Teleoperated Master–slave	Eye gaze tracking glasses, head control, joystick (insertion/withdrawal)	Research prototype (lab studies)	2021	*Surgery—minimally invasive* Endoscopic submucosal dissection (ESD)

^a^NeoGuide was acquired by Intuitive Surgical Inc., US

^b^Invendo medical was acquired by Ambu, Denmark

### User interfaces used in robot-assisted surgical platforms

**Fig. 4:**
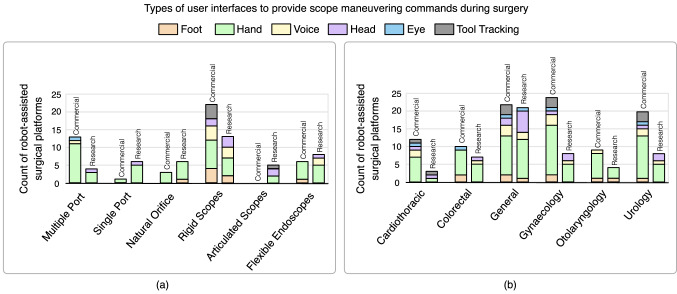
Mapping of user interfaces with robotic systems and surgeries

Robot-assisted surgical platforms presented above utilize different user interfaces for scope manipulation. Overall, the results presented in Fig. 4a and Table 7 suggest that robotic surgical systems predominantly use hand control interfaces, whereas robotic scope holders tend to utilize and experiment with a variety of different interfaces, including tool tracking. In robotic surgical systems for multiple port, single port, and natural orifice, the design of closed consoles requires the surgeon to place their head on the stereo viewer. This limits the surgeon’s range of movement, making hand controllers appropriate for scope control. Most commercially available robotic scope holders offer a hand control interface due to its familiarity and intuitiveness which is necessary while performing surgical procedures. Advantages such as user-friendliness, easy hand–eye coordination, and lower cognitive load make hand control popular.

**Table 7:** Mapping of actuated scopes with common user interfaces used

Interface	System type	Robotic surgical systems			Robotic scope holders		
		Multiple port	Single port	Natural orifice	Rigid scopes	Articulated scopes	Endoscopes
Foot	Commercial				AESOP [85] Cirq [109] HIWIN MTG-H100 [23, 107, 108] RoboLens [20, 99–101]		Endodrive [84, 134, 135]
	Research			Three-Limb Robotic System [16, 80, 81]	FREEDOM [118, 119] Yang, Udatha [17]		
Hand	Commercial	avatera [41, 42] da Vinci Xi [29, 33] Dexter [46, 47] hinotori [44, 45] Hugo RAS system [52–54] Micro hand S [34] Revo-i [37–39] Senhance [35–37] SSI mantra [55] Toumai [49] Versius [43]	da Vinci SP [36, 58]	Flex system [29, 68, 69] Ion endoluminal system [75–77] MONARCH platform [14, 70–73]	AESOP [85] AutoLap [8, 86, 102, 103] EinsteinVision 3.0 [110, 111] LapMan [88–90] Naviot [91] RoboLens [20, 99–101] ROSA ONE brain [116] SOLOASSIST II [112–115]		Aer-O-Scope [139–141] Avicenna Roboflex [136, 137] Endotics [130, 132, 133] invendoscopy E200 System [29, 142, 143] NeoGuide [128–131]
	Research	ARTEMIS [31] Bitrack [29, 40] SHURUI [27, 50, 51]	Enos [28, 37, 64, 65] MIRA [66] SPAS robotic system [62, 63] SurgiBot [30] SURS [59]	ETRS [78] *i*^*2*^*Snake* [74] K-FLEX [79] STRAS [13, 16] Three-Limb Robotic System [16, 80, 81] Endoluminal surgical system [82, 83]	Avellino, Bailly [120] De Pauw, Kalmar [117] EVOLAP [97, 98] FIPS [86, 87]	Cardioscope [121, 122] PliENT [125]	EOR [145, 146] GIM [144] RAFE [127] Sivananthan, Kogkas [147] Teleflex [10, 138]
Voice	Commercial	ZEUS [32]			AESOP [85] RoboLens [20, 99–101] SOLOASSIST II [112–115] ViKY [92–95]		
	Research				FAce MOUSe [22] FIPS [86, 87] HIWIN MTG-H100^a^ [23, 107, 108]		
Head	Commercial				EMARO [86, 104–106] FreeHand [86, 96]		
	Research	Jo, Kim [48]	Vicarious [60, 61]		Avellino, Bailly [120] FAce MOUSe [22] HIWIN MTG-H100 [23, 107, 108]	ARSFE [26, 126] Omori, Arai [123]	Sivananthan, Kogkas [147] Teleflex [10, 138]
Eye	Commercial	Senhance [35–37]					
	Research						Sivananthan, Kogkas [147]
Tool Tracking	Commercial				AutoLap [8, 86, 102, 103] Avellino, Bailly [120] RoboLens [20, 99–101] ViKY [92–95]		
	Research					ARSFE [26, 126]	

^a^Voice and head control are not present in the commercially available HIWIN MTG-H100 system

**Fig. 5:**
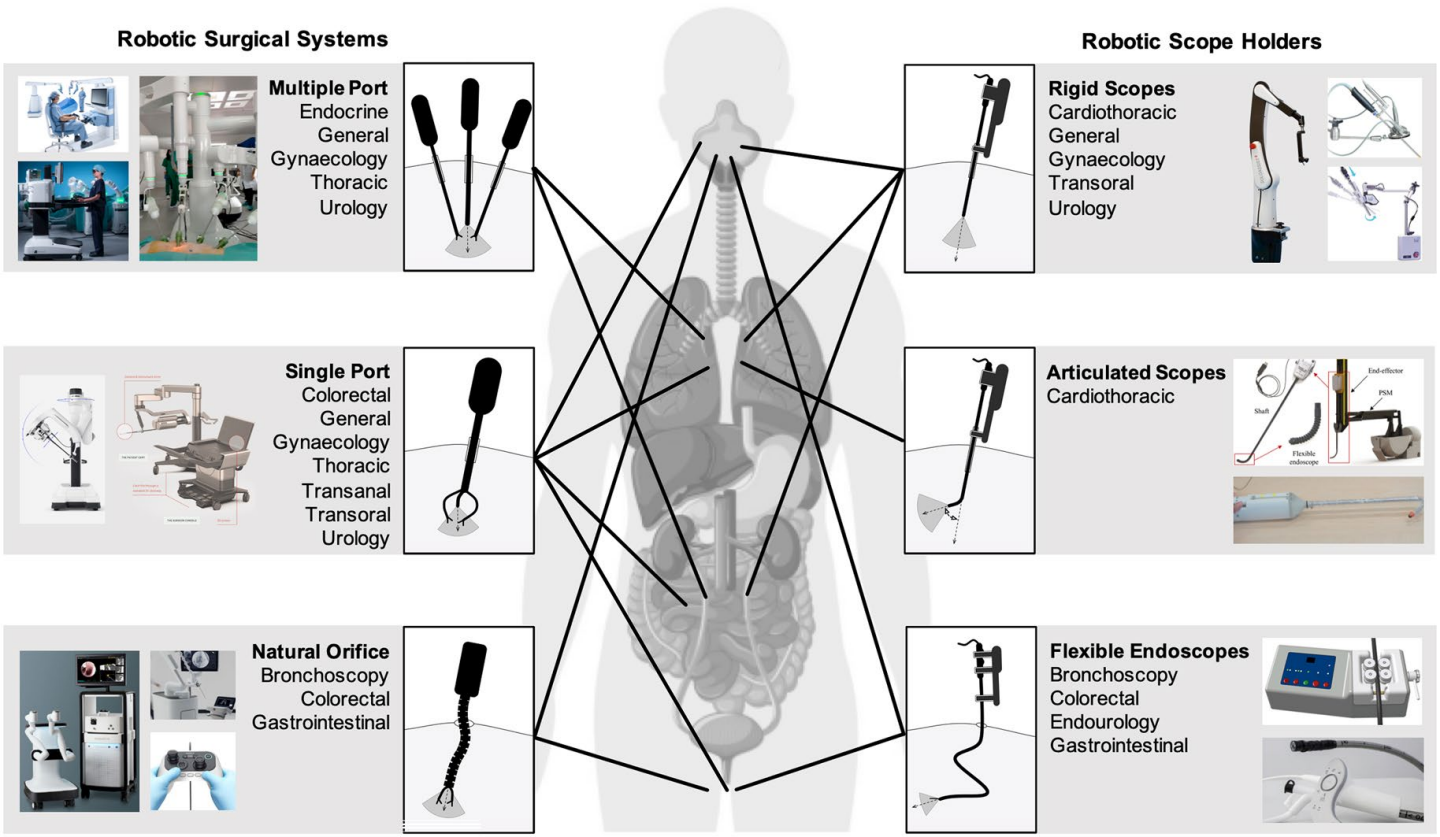
Surgical applications and entry port sites of various robotic systems

As shown in Fig. 4b and Table 8, all categories of interfaces are used in general, urology, and gynecology surgeries. Otolaryngology, which focuses on ears, nose, and throat, predominantly utilizes hand control, and has the least variety of interfaces applied. Figure 5 illustrates the key surgical applications of the robotic systems, and the entry port sites. About 85% of prostatectomies in the USA are performed using robot assistance [148]. Complexity of the procedure and surgeon’s prior experience with related technology both affect the learning curve in robotic surgery [25].

**Table 8:** Common areas of surgical specialties and the interfaces used for robotic scope control

Surgical specialty	System type	User interface for robotic scope control						
		Foot	Hand		Voice	Head	Eye	Tool
Cardiothoracic surgery Coronary artery bypass grafting (CABG) Lung cancer surgery Mitral valve repair	Commercial		da Vinci Xi [29, 33]	Naviot [91] Senhance [35–37] SOLOASSIST II [112–115] SSI Mantra [55]	SOLOASSIST II [112–115] ViKY [92–95]	FreeHand [86, 96]	Senhance [35–37]	ViKY [92–95]
	Research			Cardioscope [121, 122]		ARSFE [26, 126]		ARSFE [26, 126]
Colorectal surgery Colon resection Rectal resection Rectopexy	Commercial	Endodrive [84, 134, 135] HIWIN MTG-H100 [23, 107, 108]	Aer-O-Scope [139–141] da Vinci Xi [29, 33] Endotics [130, 132, 133]	Invendoscopy E200 System [29, 142, 143] Micro hand S [34] Senhance [35–37] Versius [43]			Senhance [35–37]	
	Research		De Pauw, Kalmar [117] EOR [145, 146]	MIRA [66] SHURUI [27, 50, 51] STRAS [13, 16] Endoluminal surgical system [82, 83]	HIWIN MTG-H100^a^ [23, 107, 108]	HIWIN MTG-H100 [23, 107, 108]		
General surgery Acid reflux disease surgery Bariatric surgery Cholecystectomy Endocrine surgery Hernia repair Liver surgery Pancreas surgery Small bowel surgery	Commercial	HIWIN MTG-H100 [23, 107, 108] RoboLens [20, 99–101]	AutoLap [8, 86, 102, 103] da Vinci Xi [29, 33] EinsteinVision 3.0 [110, 111] Micro Hand S [34] Naviot [91]	Revo-i [37–39] RoboLens [20, 99–101] Senhance [35–37] SOLOASSIST II [112–115] SSI Mantra [55] Versius [43]	RoboLens [20, 99–101] SOLOASSIST II [112–115] ViKY [92–95]	EMARO [86, 104–106] FreeHand [86, 96]	Senhance [35–37]	AutoLap [8, 86, 102, 103] RoboLens [20, 99–101] ViKY [92–95]
	Research	Three-limb robotic system [16, 80, 81]	Bitrack [29, 40] Enos [28, 37, 64, 65] EOR [145, 146] ETRS [78] GIM [144] K-FLEX [79]	RAFE [127] Sivananthan, Kogkas [147] SurgiBot [30] Teleflex [10, 138] Three-Limb Robotic System [16, 80, 81]	FAce MOUSe [22] HIWIN MTG-H100 [23, 107, 108]	FAce MOUSe [22] HIWIN MTG-H100 [23, 107, 108] Omori, Arai [123] Sivananthan, Kogkas [147] Teleflex [10, 138] Vicarious [60, 61]	Sivananthan, Kogkas [147]	
Gynecology Endometriosis resection Hysterectomy Myomectomy Ovarian cystectomy Pelvic organ prolapse surgery	Commercial	HIWIN MTG-H100 [23, 107, 108] RoboLens [20, 99–101]	AutoLap [8, 86, 102, 103] avatera [41, 42] Avicenna Roboflex [136, 137] da Vinci Xi [29, 33] Dexter [46, 47] Flex System [29, 68, 69]	Hugo RAS system [52–54] LapMan [88–90] Revo-i [37–39] RoboLens [20, 99–101] Senhance [35–37] SOLOASSIST II [112–115] SSI Mantra [55] Versius [43]	RoboLens [20, 99–101] SOLOASSIST II [112–115] ViKY [92–95]	FreeHand [86, 96]	Senhance [35–37]	AutoLap [8, 86, 102, 103] Avellino, Bailly [120] RoboLens [20, 99–101] ViKY [92–95]
	Research		Avellino, Bailly [120] Bitrack [29, 40] Enos [28, 37, 64, 65]	EVOLAP [97, 98] SHURUI [27, 50, 51]	HIWIN MTG-H100 [23, 107, 108]	Avellino, Bailly [120] HIWIN MTG-H100 [23, 107, 108]		
Otolaryngology Sinus surgery Surgery for tumors in mouth and throat Tongue base resection	Commercial	Cirq [109]	da Vinci SP [36, 58] da Vinci Xi [29, 33] Flex System [29, 68, 69]	ROSA ONE Brain [116] SOLOASSIST II [112–115] SSI Mantra [55] Versius [43]	SOLOASSIST II [112–115]			
	Research	FREEDOM [118, 119]	*i*^*2*^*Snake* [74] K-FLEX [79]	PliENT [125]				
Urology Bladder surgery Cyst removal Kidney surgery Prostate surgery Pyeloplasty Ureteral implantation	Commercial	HIWIN MTG-H100 [23, 107, 108]	AutoLap [8, 86, 102, 103] avatera [41, 42] da Vinci SP [36, 58] da Vinci Xi [29, 33] hinotori [44, 45] Hugo RAS System [52–54]	Revo-i [37–39] Senhance [35–37] SOLOASSIST II [112–115] SSI Mantra [55] Toumai [49]	SOLOASSIST II [112–115] ViKY [92–95]	FreeHand [86, 96]	Senhance [35–37]	AutoLap [8, 86, 102, 103] Avellino, Bailly [120] ViKY [92–95]
	Research		Avellino, Bailly [120] Bitrack [29, 40] SHURUI [27, 50, 51]	SPAS Robotic System [62, 63] SurgiBot [30]	HIWIN MTG-H100 [23, 107, 108]	Avellino, Bailly [120] HIWIN MTG-H100 [23, 107, 108]		

^a^Voice and head control are not present in the commercially available HIWIN MTG-H100 system.

## Discussion

**Fig. 6:**
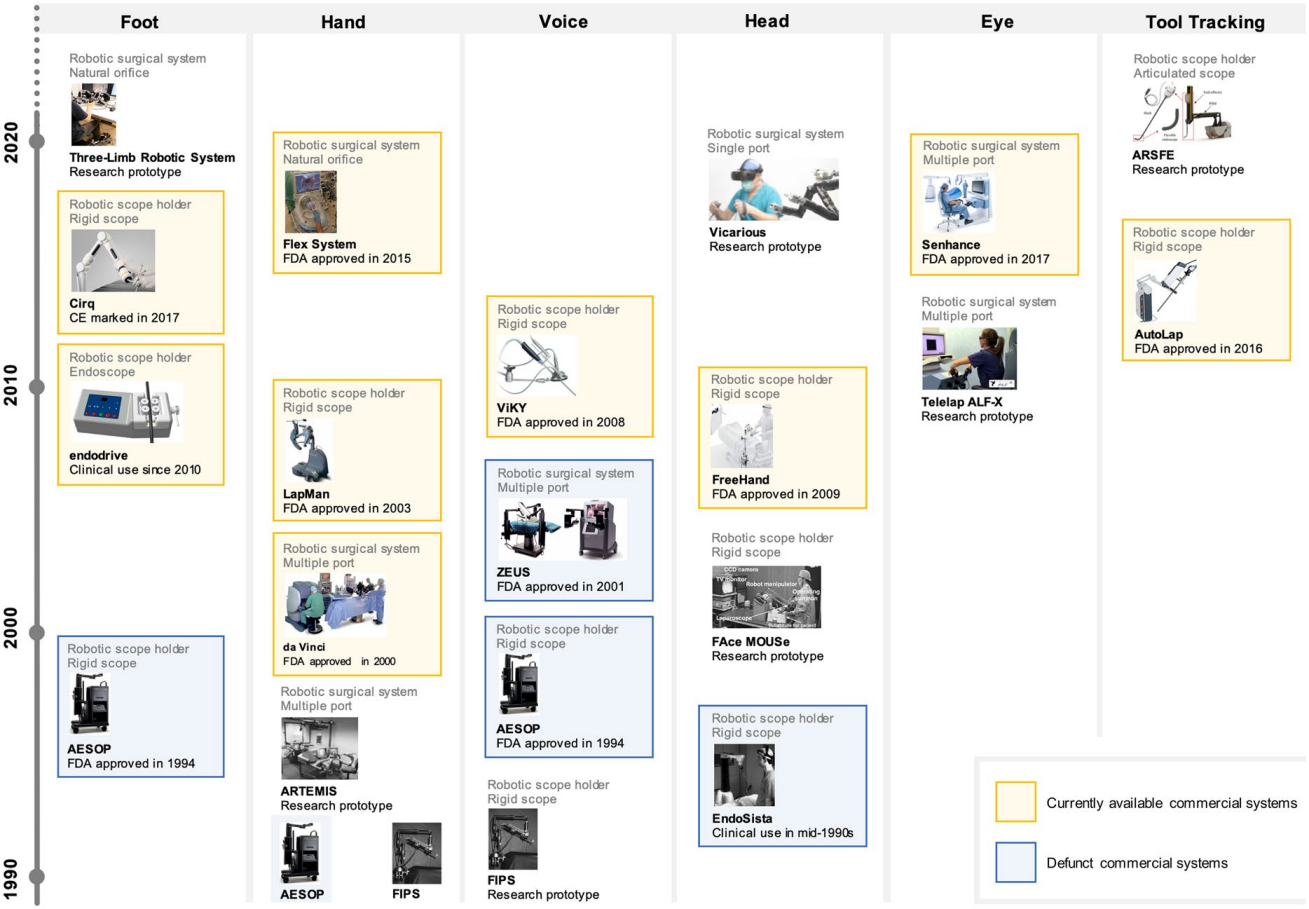
Early appearances of different user interfaces in research and commercial robotic systems

Use of robot assistance in surgeries has increased in the past decade. Early appearances of user interfaces in research and commercial robotic systems are illustrated in Fig. 6. In the period of 1990–2010, commercial systems were chiefly controlled using foot, hand, voice, and head interfaces, while the period of 2010–2020 has witnessed the emergence of eye-gaze and tool tracking scope control interfaces. AESOP and ZEUS systems (Computer Motion Inc., USA) developed during the mid to late 1990s both utilized voice commands as input [32], mimicking the default communication between surgeon and assistant. Computer Motion Inc. was acquired by Intuitive Surgical which uses hand interfaces for their da Vinci systems. Intuitive Surgical has been the market leader since early 2000s [149]. Head motion for rigid scope control was first used in EndoSista (Armstrong Healthcare, UK) during the mid-1990s [150]. It was later commercialized by FreeHand Surgical, UK in 2008. Tool tracking, as implemented in the AutoLap system (MST Medical Surgery Technologies, Israel) in 2016, has received more attention recently.

There has been a limited number of studies comparing different user interfaces. These studies focus on robotic scope holders for rigid scopes. A summary of these studies is presented in Table 9, which illustrates that surgeons increasingly prefer scope control interfaces that free their hands to control surgical instruments and do not interrupt surgical tasks. Voice control was favored due to its reduced length of operating time and improved concentration [151]. However, foot control was preferred in multiple studies. In studies [19–21] comparing foot and voice controls that keep surgeon’s hands free, foot control was preferred, as voice commands had a higher chance of misinterpretation. In addition to task completion time, Allaf, Jackman [19] measured operator-interface failures, which was defined as occasions where the surgeon had to focus attention on the interface rather than the surgical field. The protocol was also repeated to assess the percentage of improvement retained after two weeks, where foot control was found easier to learn. While comparing AESOP and ViKY systems [21], it was found that voice commands had to be repeated due to speech recognition failures. Voice control was found to be affected by pronunciation while evaluating the RoboLens [20]. The system was assessed based on time for procedure completion, need for cleaning, image stability, and procedure field centering during several laparoscopic cholecystectomies. A significant lag between voice command and scope movement was observed. Although foot control is preferred over voice, eye–foot coordination might not be ideal, and surgeons often looked down to choose the right pedal from multiple ones [151]. Tool tracking is increasingly preferred as there is no interruption to surgery to control the scope. In a study by Avellino et al. [120] comparing joystick controlled by hand, body posture tracking and tool tracking, surgeons evaluated the interfaces based on a defined set of tasks. Joystick received good ratings and was ranked behind tool tracking, while posture tracking was found suitable for tasks requiring short distance movements. Despite raising concerns for tasks that do not involve surgical instruments, tool tracking was well-regarded.

**Table 9:** Comparison of different interfaces for scope control, by year

Study & year	Interfaces	Robotic system	Comparators	Observation
Allaf, Jackman [19] (1998)	Voice & foot	AESOP 2000	Mean task completion time, operator-interface failure per trial, & durability of learning experience retained over two weeks	Foot control was preferred over voice Voice commands were misinterpreted, whereas foot control was quick & easier to learn (*p* < 0.002)
Mettler, Ibrahim [151] (1998)	Feet, hand & voice	AESOP 2000	Operating time length	Voice control was favored over foot & hand controls Foot pedal was preferred over hand control as it freed surgeon’s arms
Berkelman, Cinquin [18] (2005)	Voice & hand	ViKY	Surgeon’s evaluation of user commands	Voice command was preferred over keypad mounted on scope
Gumbs, Crovari [21] (2007)	Voice & foot	ViKY & AESOP 3000	Average setup time, repetition of commands, occurrence of errant commands	Foot pedal was preferred over voice by surgeons Voice commands were likely to be misheard, while there was no chance for misinterpreted commands with foot pedals. Better setup and removal time was observed for ViKY (*p* < 0.001)
Mirbagheri, Farahmand [20] (2011)	Voice & foot	RoboLens	Procedure completion time, need for cleaning, image stability, procedure field centering, surgeon’s evaluation of interface	Foot control was preferred over voice by surgeons Voice recognition was affected by pronunciation, and significant lag was observed between voice command and scope movement
Kranzfelder, Schneider [152] (2014)	Feet, voice & eye	–	Surgeon’s evaluation	As an addition to hand control, foot pedal was preferred over speech and eye tracking by surgeons & gastroenterologists (56%). More specialists preferred foot control than generalists
Avellino, Bailly [120] (2020)	Hand (joystick), body posture & tool tracking	–	Surgeon’s evaluation of stability, precision, cognitive load, and intuitiveness as criteria	Tool tracking was preferred Posture tracking may be considered for tasks that require short distance movement

Overall, actuated scopes utilize a variety of user interfaces such as foot, hand, voice, head, eyes, and tool tracking to provide stable views and smooth control during minimally invasive surgeries. Hand control is the most popular interface across all categories of surgical systems as it is familiar, intuitive and requires less mental load. However, various other interfaces are being investigated to address the interruption to surgical workflow caused by hand control. Head tracking interfaces are being explored in research prototypes such as the multiple-port system by Jo et al. [48]. This helps address the issue of interruption to surgical procedure caused by hand interfaces when switching control between surgical instrument and scope. Breaks in surgical workflow can result in longer operating time and increased risk of patient injury [48]. Having an easy-to-use and intuitive single-person interface is considered important for scope control by surgeons and gastroenterologists [152]. In teleoperated systems, where the surgeon is away from the patient, there is a preference for an open surgeon console. In an open console design, the surgeon views the video feedback through a head-up display, as opposed to an enclosed stereo viewer. Compared to a closed console, an open platform offers increased situational awareness, enables the expert surgeon to effectively mentor interns, and improve team communication [153, 154]. Preference for working position, either sitting or standing, varies among surgeons [152].

Majority of the systems utilizing hand controllers (such as da Vinci—Intuitive Surgical, Revo-i—Revo Surgical Solutions, and Enos—Titan Medical) or head-motion-based controllers (such as FreeHand system and MTG-H100–HIWIN) requires a foot pedal to activate the scope control mechanism. In these multimodal user interfaces, the foot pedal has two functionalities. First, it acts as an on–off switch that triggers the motion of the scope. In case of hand controllers, it enables the operator to switch the control from surgical instruments motion (to operate on the tissue) to scope maneuvering (to navigate the operative field). In case of head-motion-based controllers, it activates the scope motion only when the foot pedal is pressed and thus allows the surgeons to freely move the head during the rest of the procedure [155, 156]. Second, the foot pedal acts as a clutch and facilitates ergonomic repositioning of the hand controllers or head position [157]. Another example of a multimodal user interface for scope control is head-mounted display (HMD) devices. HMDs have been used in the operating room for surgical navigation and planning [158, 159]. In case of actuated scope maneuvering, the operative field view is rendered by HMD devices in a virtual reality or a mixed reality environment, whereas head motions detected by the device’s sensors are used to maneuver the scope [160–162]. In contrast to visualizing the operative field on a physical screen, the usage of HMD devices offers the surgeon the flexibility to ergonomically place the virtual view of the operative field in the operating room [5, 163, 164]. It decreases the surgeon’s shift of focus from the screen to the operating site [165, 166] and thus may assist in reducing the prolonged strains (in the neck and lower back) due to bad monitor positioning [167, 168]. Further end-user clinical studies would be required to assess the potential of HMD devices as a multimodal user interface (i.e., to immerse the operator with the information pertaining to the operating field and evaluate the control of the robotic system [169, 170]).

Limitations of this review include removal of non-English literature. The exclusion may have prevented a broad representation and insight. Methodological quality of the included studies was also not assessed. Additionally, there are no studies comparing all the different user interfaces with the same surgical task and scenario, which would have provided an equal assessment.

In conclusion, the observations in this review indicate that integration of multiple control interfaces for camera control would be ideal, especially for scope holders used in bed-side procedures. As each interface has its own benefits, merging different control types enables the surgeon to benefit specifically from each interface in various surgical steps [120]. The surgeon would be free to choose the appropriate control type throughout different stages of the surgical procedure. Integration of head tracking, which is efficient for 3D navigation, or tool tracking, which lowers cognitive load, would be advantageous. Nevertheless, merging several controls may result in limitations such as redundancy. It may also pose a challenge for the surgeon to achieve seamless transition while changing interfaces. It would be helpful to further explore the impact of different user interfaces on surgical outcomes in future studies.

## Supplementary Information

Below is the link to the electronic supplementary material.

Supplementary file1 (DOC 73 kb)
